# Human Haemato-Endothelial Precursors: Cord Blood CD34+ Cells Produce Haemogenic Endothelium

**DOI:** 10.1371/journal.pone.0051109

**Published:** 2012-12-04

**Authors:** Elvira Pelosi, Germana Castelli, Ines Martin-Padura, Veronica Bordoni, Simona Santoro, Alice Conigliaro, Anna Maria Cerio, Marco De Santis Puzzonia, Paola Marighetti, Mauro Biffoni, Tonino Alonzi, Laura Amicone, Myriam Alcalay, Francesco Bertolini, Ugo Testa, Marco Tripodi

**Affiliations:** 1 Department of Haematology, Oncology and Molecular Medicine, Istituto Superiore di Sanità, Rome, Italy; 2 L. Spallanzani National Institute for Infectious Diseases, IRCCS, Rome, Italy; 3 Istituto Pasteur-Cenci Bolognetti, Dipartimento di Biotecnologie Cellulari ed Ematologia, Università Sapienza, Rome, Italy; 4 Laboratory of Haematology-Oncology, Department of Medicine, European Institute of Oncology, Milan, Italy; 5 Department of Experimental Oncology, Istituto Europeo di Oncologia, Milan, Italy; 6 Dipartimento di Medicina, Chirurgia e Odontoiatria, Universit? degli Studi di Milano, Milan, Italy; University of Udine, Italy

## Abstract

Embryologic and genetic evidence suggest a common origin of haematopoietic and endothelial lineages. In the murine embryo, recent studies indicate the presence of haemogenic endothelium and of a common haemato-endothelial precursor, the haemangioblast. Conversely, so far, little evidence supports the presence of haemogenic endothelium and haemangioblasts in later stages of development. Our studies indicate that human cord blood haematopoietic progenitors (CD34+45+144−), triggered by murine hepatocyte conditioned medium, differentiate into adherent proliferating endothelial precursors (CD144+CD105+CD146+CD31+CD45−) capable of functioning as haemogenic endothelium. These cells, proven to give rise to functional vasculature *in vivo,* if further instructed by haematopoietic growth factors, first switch to transitional CD144+45+ cells and then to haematopoietic cells. These results highlight the plasticity of haemato-endhothelial precursors in human post-natal life. Furthermore, these studies may provide highly enriched populations of human post-fetal haemogenic endothelium, paving the way for innovative projects at a basic and possibly clinical level.

## Introduction

The origin(s) of vascular and blood cell types during development is not entirely clear and may be different depending on the stage of hematopoiesis and the site of blood cell development.

During primitive hematopoiesis, the earliest stage of blood development, hematopoietic and endothelial cells emerge simultaneously. Their origin is highly debated: if, as it has been proposed, they are issued independent of the differentiation of mesodermal stem/progenitor cells [1] or, according to an alternative view, they derive from a common bi-potent progenitor called the hemangioblast [2], [3]. Evidence supporting the transient existence of the haemangioblast was first provided by *in vitro* differentiation of embryonic stem cells [4]; haemangioblasts have also been isolated in the avian caudal mesoderm [5], as well as in mouse [2] and zebrafish [6] embryos, and human cord blood (CB) CD34+ cells, specifically in the CD34+KDR+ subpopulation [7] and CD34+133+ subpopulation [8].

Primitive hematopoietic activity is eventually supplanted by the second wave of multilineage (definitive) hematopoiesis. Pluripotent hematopoietic stem cells (HSCs) and multipotent hematopoietic progenitor cells (HPCs) are considered to be issued from specialized endothelial cells, commonly defined as haemogenic endothelium. While the existence of hemangioblastic cells *in vivo*, as well as their contribution to the development of primitive hematopoiesis remains controversial, growing evidence supports the concept that multipotent HSCs and multilineage HPCs arise from haemogenic endothelium (for review: [9]). Embryos of many vertebrates show transient haemogenesis on the aortic endothelium floor, designated as haemogenic endothelium [10], [11]. The haemogenic endothelium model is further supported by studies showing that HPCs of both embryonic and yolk sac origin differentiate from VE-cadherin+ ECs. [12], [13], [14] Similarly, HSC and HPCs are generated in the placental vasculature, apparently through haemogenic differentiation of placental endothelium. [15] Elegant experiments based on VE-cadherin lineage tracing and single-cell imaging provide definitive evidence for the endothelial origin of haematopoietic cells in ES culture [16], as well as in zebrafish [17], [18] and mouse [19] embryos. Notably, other studies suggested that the haemangioblast generates haematopoietic cells through a haemogenic endothelium intermediate stage [20].

However, is currently unknown whether haemogenic endothelium contribute to the development of hematopoiesis, also at later stages of development (i.e. whether haemogenic endothelium exist in human cord blood and bone marrow). Some observations suggest a possible contribution of haemogenic endothelium to the generation of HSCs during adult life. In fact, Zovein et al. [11] through embryonic lineage-tracing studies, which label haemogenic endothelium before definitive hematopoiesis and follow their progeny HSCs during adult life, demonstrate that although embryonic HSCs remain functional in the adult, they do not account for all hematopoietic cells generated postnatally. Furthermore, Wu et al. [8] provided preliminary evidence that human CD34+ cord blood cells, when grown in appropriate cell culture conditions, could generate an endothelial cell progeny endowed with properties of haemogenic endothelium.

We previously described [21] the influence of soluble factors in conditioned medium released by murine hepatocyte conditioned medium (MH-CM) on human CB CD34+ progenitors; in long- term MH-CM culture we obtained growth of: (i) a bulk CD34+ population differentiating toward the endothelial lineage and (ii) single CD34+ cells expressing both haematopoietic (CD45) and endothelial (CD144) markers.

In the present study, we explored the potential of human CB CD34+ HPCs to differentiate into haemogenic endothelium. In long-term culture, the addition of MH-CM stimulates the initial CD34+45+144− HPCs to generate adherent CD45−144+ endothelial precursors capable of self-renewal/proliferation and to differentiate in endothelial cells *in vivo*. These cells, instructed by haematopoietic growth factors (HGFs), rapidly differentiate into CD45+144+ cells that in turn generate either (i) CD45+ haematopoietic cells (mainly of erythroid and megakaryocytic type) when grown in haematopoietic medium, or (ii) CD144+ ECs if cultured in endothelial medium.

## Results

### Cord Blood CD34+ Cell Cultures in MH-CM Medium Allow for the Appearance of Endothelial Progenitors

**Figure 1:**
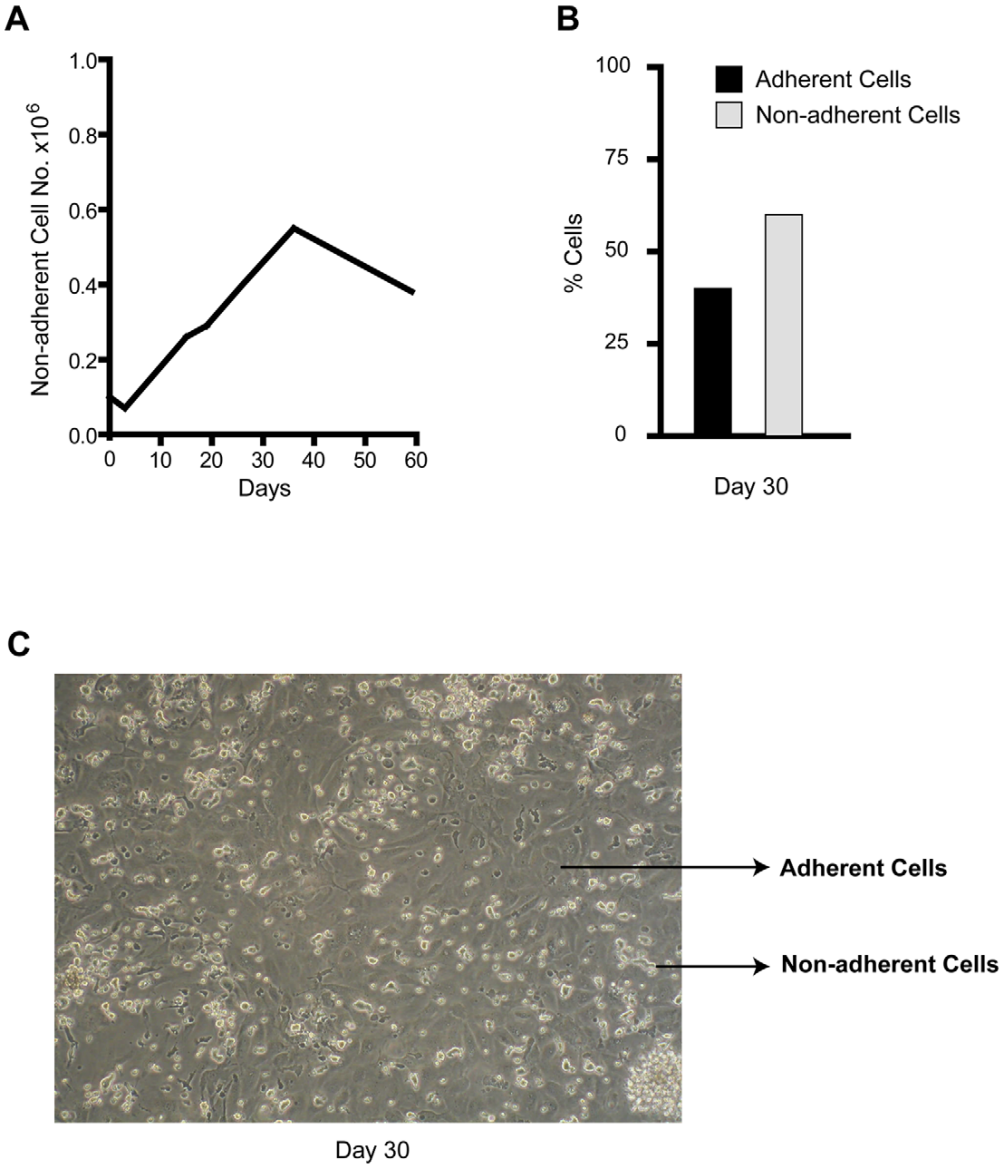
Characterization of CB CD34+ cells grown in MH-CM culture. A- Growth curve of cells grown in MH-CM; a representative experiment out of 10 is shown. B- Percentage of adherent and non-adherent cells grown in MH-CM at day 30; a representative experiment out of 10 is shown. C- Phase-contrast morphology of day 30, adherent and non-adherent cells (original magnification 10x). Non-adherent cells are round and more refractive, while adherent cells are flat and less refractive; a representative experiment out of 10 is shown.

We previously described the influence of MH-CM on CB derived CD34+45+144− HPCs. Specifically, MH-CM allowed for long-term culture of proliferating CD34+ cells, which gradually gave rise to two different populations: i) one floating and round shaped, composed of CD45+ haematopoietic cells and ii) one firmly attached to the wells, which acquired endothelial morphology and became CD144+ [21]. Figure 1 recapitulates these results showing the expansion of the CB CD34+ cell derived progeny cultured in MH-CM up to 60 days, and the cellular morphology at day 30, when the floating and adherent cell subpopulations are clearly distinguishable.

From these observations, we further characterized the CD34+ derived adherent cells fraction at both phenotypic and functional levels.

**Figure 2:**
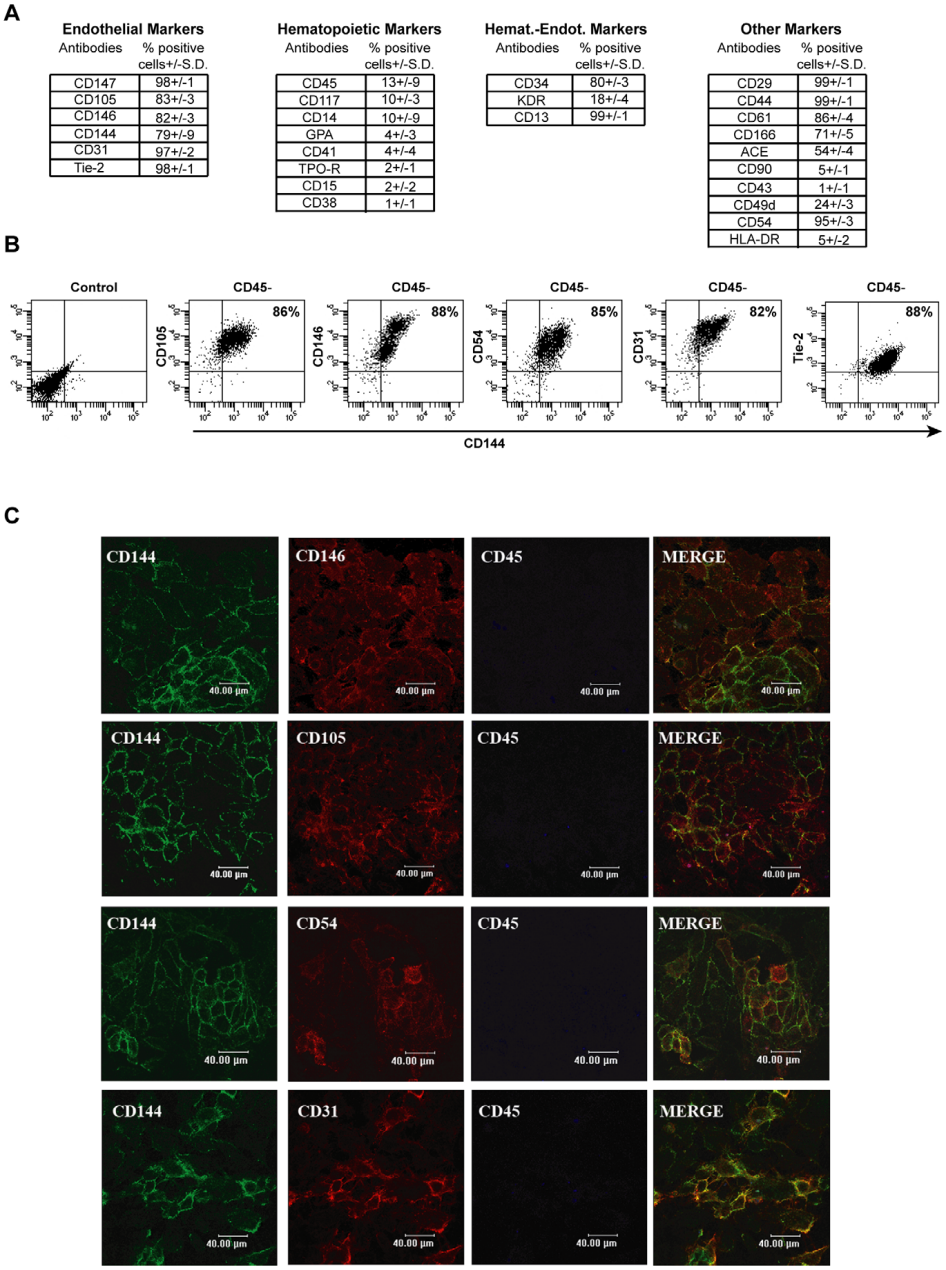
Phenotypic characterization of adherent cells in MH-CM culture. A- Immunophenotypic characterization of adherent cells generated by CD34+ cells cultured in MH-CM for 30 days. Mean values +/− SD of 15 independent experiments are shown. B- Flow cytometry analysis of the indicated endothelial markers expressed by adherent cells generated by CD34+ cells cultured in MH-CM for 30 days. Cells have been labelled with CD45, CD144 and with the indicated antigen. Shown are plots of CD45 negative cells (87+/−9%). The percentage of double-positive cells for the two endothelial antigens are given. A representative experiment out of 15 is shown. C- Confocal imaging: triple immunostaining analysis for the indicated antigens of adherent cells generated by CD34+ cultured in MH-CM for 30 days. A representative experiment out out of 5 is shown.

Firstly, CD34+ derived adherent cell progeny, cultured in MH-CM for 30 days and isolated by washing away the floating cells, was phenotypically characterized using a broad range of different cell-surface markers. As shown in Figure 2A the majority of cells were positive for: CD144, CD146, CD147, CD105 (endothelial markers), CD34 (haemato-endothelial marker), CD29, CD44 (mesenchymal markers) CD61, i.e. β3 integrin-GPIIIa (megakaryocytic-endothelial-osteoclastic marker) and CD13, i.e. aminopeptidase N (myeloid-endothelial marker). On the contrary, these cells were found largely negative for other haematopoietic markers, including CD45, Glycophorin-A (GPA, erythroid marker), CD15 (myeloid marker) CD41 and TPO-R (megakaryocytic markers). Since, no univocal endothelial markers have been identified [22] to date, the endothelial nature of the adherent cells was better characterized by multiple markers analysis: the expression of CD45 and CD144 was analyzed in combination with other endothelial markers (i.e. CD146, CD105, CD31, CD54 and Tie2). This characterization by triple labeling demonstrated that the great majority of the CD45−CD144+ adherent population co-expresses all of these markers (Fig. 2B). Notably, these results were confirmed by confocal analysis of the adherent cell population. As shown in Figure 2C, the adherent cells co-express endothelial antigens, while they are negative for CD45. Altogether this immunophenotypic analysis supports the endothelial nature of the CD34+ derived adherent cells.

**Figure 3:**
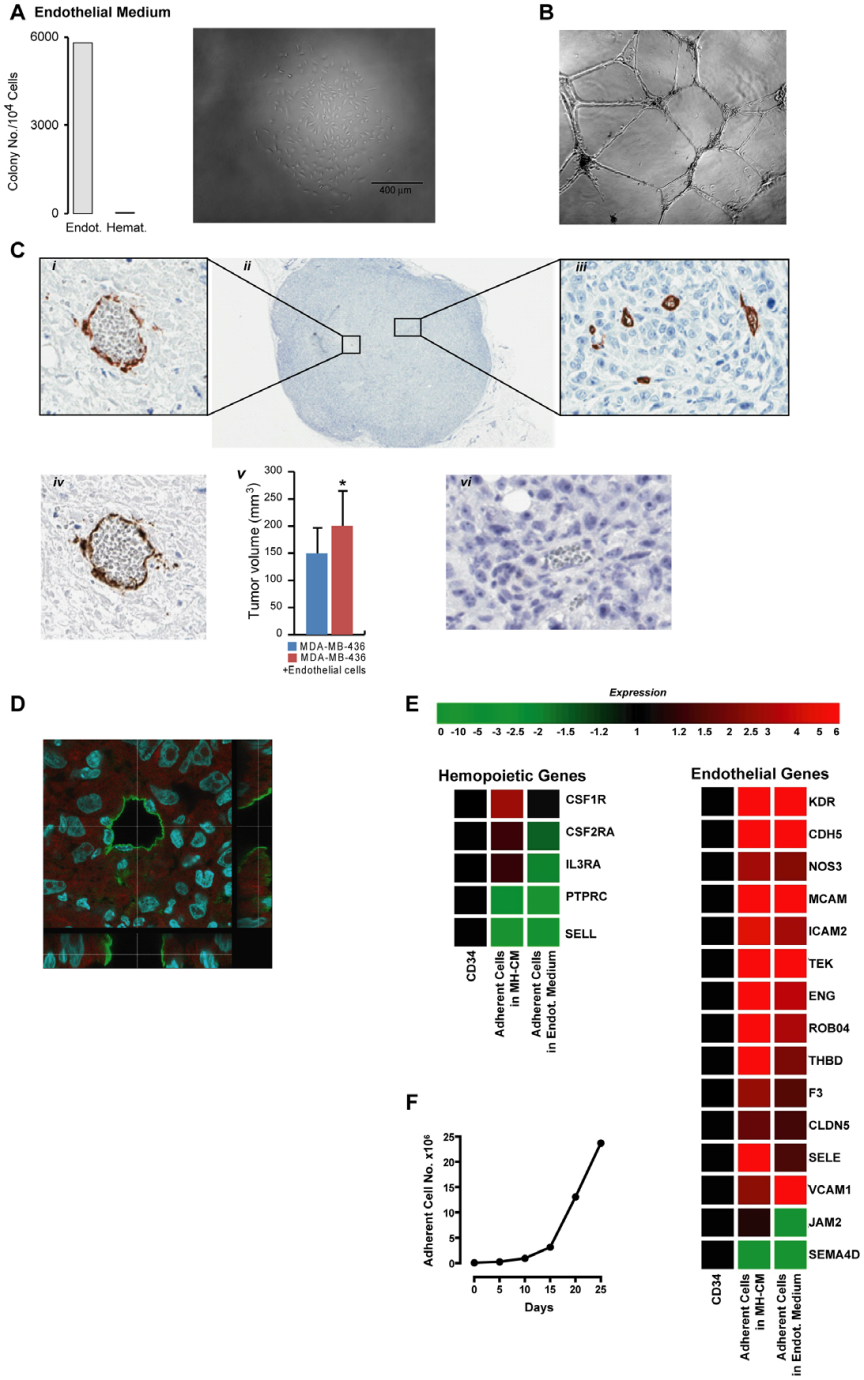
Functional characterization of adherent cells in MH-CM culture. A- *left panel* -Clonogenetic assay of adherent cells generated by CD34+ cultured in MH-CM for 30 days, carried out in semi-solid culture supplemented with the indicated medium. *Right panel* - Light microscopy image of a single cell-derived colony grown in semi-solid EGM2 endothelial medium. A representative experiment out of 5 is shown. B-Phase-contrast morphology of adherent cells cultured on matrigel supplemented with EGM-2 for 12 hours (original magnification 10x). A representative experiment out of 5 is shown. C- Immunohistochemistry evaluation of adherent cells, generated by CD34+ cultured in MH-CM for 30 days, engrafted in breast cancer-bearing NSG mice (n = 5). Human CD34 (i,ii,iii) and human CD31 (iv) detect functional vessels containing red blood cells. These antibodies are human-specific and do not cross react with mouse vessels (vi). A representative experiment out of 5 is shown. (v) Tumor volume quantification showing that the volumes of tumors where breast cancer cells were co-injected with the adherent cells generated by CD34+ cells are significantly higher (* p<0.04) than the volume of tumors where breast cancer cells alone were injected in NSG mice (n = 5/group). D-Confocal laser-scanning of human CD34 antigen distribution in a tumor section of a MB-MDA436 plus adherent cells, generated by CD34+ cultured in MH-CM for 30 days. Images were acquired using a Leica TCS SP5 confocal microscope and sequential Z-stacks were performed using a 63×1.4NA oil immersion objective, zoom 3X, 0.3 µm z step. Snapshot images are orthogonal sections of the z-stacks taken at points along the vessel’s cavity. A representative picture of one tumor out of 5 is shown. E- Expression levels of haemopoietic and endothelial marker genes obtained from microarray analysis of fresh CB CD34+ cells (CD34), the adherent cellular progeny obtained after 53 days of culture in MH-CM (Adherent Cells in MH-CM) and this adherent cellular progeny grown in endothelial medium (EGM2) for additional 7 days (Adherent Cells in Endot. Medium). Expression values were normalized using CD34+ cells as control. A global up-regulation of endothelial marker genes was observed in adherent cells grown in MH-CM, both prior to and after instruction by EGM2, whereas haemopoietic markers were either unvaried or down-regulated. A representative analysis out of 3 is shown. F- Growth curve of the adherent cellular progeny obtained from CB CD34+ cells grown in MH-CM when transferred into EGM2 endothelial medium. A representative experiment out of 10 is shown.

Next, we aimed to characterize the adherent population at functional and transcriptional levels. The day-30 adherent subpopulation, when seeded in endothelial clonogenic conditions, was found to give rise to endothelial colonies, (efficiency ∼60%) (Figure 3A). Their further characterization *in vitro and in vivo* demonstrated that when cultured on matrigel these cells form tubuli (Figure 3B), the *in vivo* experiments most notably demonstrated their nature of endothelial progenitors. In fact, Figures 3D and 3E show the capacity of these cells to incorporate into functional, blood -containing, newly forming vasculature, and sustain tumor growth *in vivo*.

Transcriptional analysis illustrates how these cells express a coherent and broad repertoire of endothelial genes. Moreover, their further growth in endothelial medium (EGM2) resulted in long-term proliferation with a gene expression profile further supporting their endothelial nature (3F and 3G).

Overall, the adherent population, arising from the long term CD34+ culture in MH-CM, generates a cell population with a defined immunophenotypic, transcriptional profile, and that possesses the functional characteristics of endothelial precursors.

### The CD34+ Derived EPCs, Instructed by HGFs, Differentiate into CD144+45+ Cells and, Eventually, into Haematopoietic Cells

Since, as reported above, the appearance of the adherent endothelial precursor population in long-term MH-CM cultures of CD34+ cells is associated with the presence of non- adherent hemopoietic cells, we hypothesized that the adherent cells may contribute not only to the long- term maintenance of haematopoietic precursors but also to its generation. We reasoned that instructive signals mediated by specific cytokines may unveil a haematopoietic potential of the endothelial precursor population.

**Figure 4:**
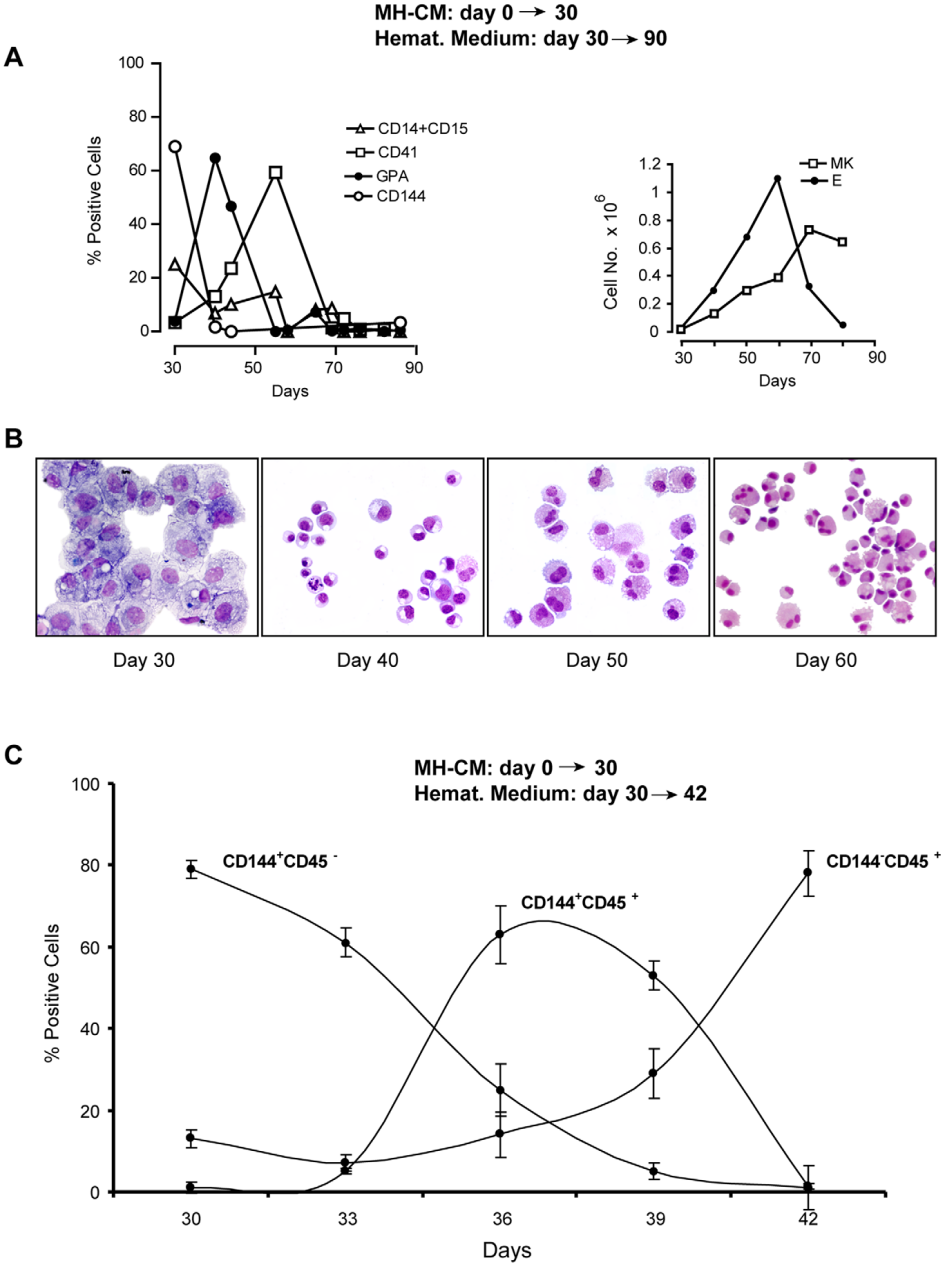
Immunophenotypic and morphological analysis of adherent ECs progeny following transfer into haematopoietic culture. A**-** Left: Time course FACS analysis of adherent cells generated by CD34+ cells cultured in MH-CM and then transferred, at day 30, to haematopoietic medium and characterized for lineage -specific antigen expression. A representative experiment out of 5 is shown. - Right: Growth curve of erythroid (E) and megakaryocytic (Mk) cells generated from the culture of day- 30 adherent cells in haematopoietic medium. A representative experiment out of 5 is shown. B**-** Morphological analysis of adherent cells generated by CD34+ cells cultured in MH-CM for 30 days, then transferred to haematopoietic medium and grown for additional days (day 30 = day 0 in haematopoietic medium, day 40 = day 10 in haematopoietic medium, day 50 = day 20 in haematopoietic medium, day 60 = day 30 in haematopoietic medium). At day 40, the large majority of cells had a morphology typical of the erythroid lineage elements at various stages of maturation. At later days of culture (day 50 and 60), erythroid cells were replaced by a cell population with a morphology compatible with the cord blood derived megakaryocytes (i.e. showing limited capacity of polyploidization). Pictures of a representative experiment out of 3 are shown. C**-** CD144 and CD45 expression analysis of adherent cells generated by CD34+ cells cultured in MH-CM for 30 days, then transferred to haematopoietic medium (i.e. day 33 = day 3 in haematopoietic medium, day 36 = day 6 in haematopoietic medium and so on). The percentage of CD144+45−, CD144+45+ and CD144−45+ cells from 8 independent experiments is reported (mean values ±SEM ).

To challenge this hypothesis, the adherent cells, generated for 30 days in MH-CM culture, were seeded into multilineage haematopoietic medium in **long-term** experiments. Notably, the adherent cells gave rise to a population of erythro-megakaryocytic cells seemingly generated by CD144+ EPCs. Specifically, we observed the appearance of erythroid cells (peaking at day 10–20 of culture in haematopoietic medium) and subsequently of megakaryocytic elements (peaking at day 30–40), as revealed by immunophenotypic (Figure 4A, left panel) and morphological (Figure 4B) analysis; the CD14+CD15+ granulo-monocytic cells only represent a minority of the generated progeny. Notably, this dramatic phenotype switch is associated with significant cell expansion (Figure 4A, right panel). Similar results were obtained using adherent cells obtained after 60 days in MH-CM culture (not shown).

These data suggest that in MH-CM culture, the adherent EPC population has the capacity to differentiate toward haemopoietic lineages, particularly the erythro-megakaryocytic ones: this may imply that these cells are reminiscent of embryonic cells previously described as haemogenic endothelium.

To further investigate the suggested differentiation capacity of the adherent EPCs toward haematopoiesis (see above), we studied the changes occurring in these cells after culture in haematopoietic medium in **short-term** experiments. Specifically, we first monitored the adherent cells for the expression of endothelial-specific (CD144) and haematopoietic-specific (CD45) markers throughout the initial 12 days of culture in haematopoietic medium. The results showed that the haematopoietic medium rapidly induces the conversion of the CD144+ cell population into CD45+ haematopoietic cells (Figure 4C). In fact, the majority of adherent cells, initially CD144+45−, became CD144+45+ within ∼5 days in haematopoietic medium culture. Subsequently, the CD144+45+ cell population rapidly declined and it was replaced by ∼80% CD144−45+ cells, within ∼7 days of culture.

**Figure 5:**
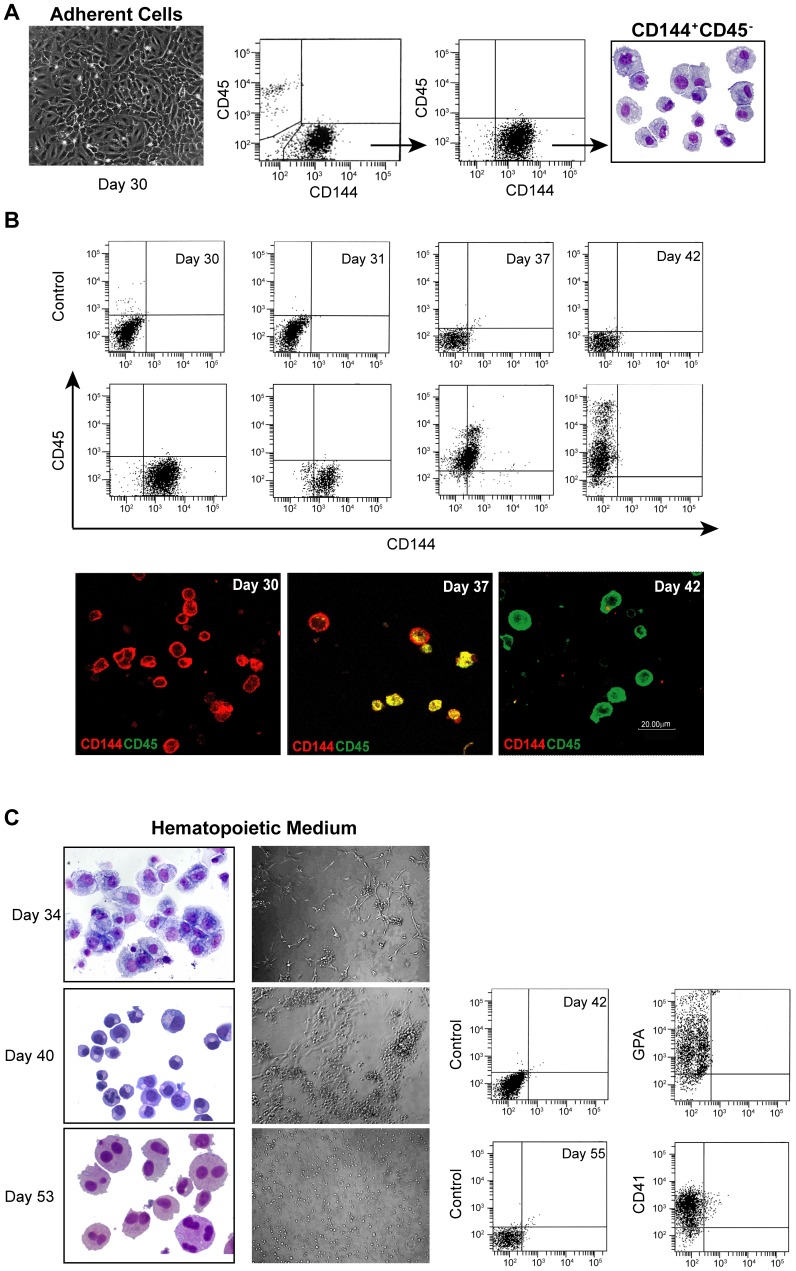
Immunophenotypic and morphological analysis of sorted CD144+45− following transfer into haematopoietic culture. A- *Left:* Phase-contrast morphology of total adherent cell population generated by CD34+ cultured in MH-CM for 30 days., *- Right*: Plots of pre-sorting and CD144+45− sorted population and morphology of post-sorted adherent CD144+45− cells at day 30 of culture. Cells were stained with MGG and analyzed by standard microscopy (40× magnification). A representative experiment out of 10 is shown. B- *Top:* Time course immunophenotypic analysis of sorted CD144+45− cells for CD144 and CD45. Controls show autofluorescences at each day of analysis. *- Bottom:* Confocal double immunofluorescence analysis for CD45 (green) and CD144 (red) antigen expression in CD144+/45− cells undergoing haemogenic differentiation at the indicated days in haemopoietic medium. A representative experiment out of 10 is shown. C- *Left*: MGG staining and phase-contrast morphology of sorted CD144+45− cells grown in haemopoietic medium for the indicated time. *- Right*: Immunophenotypic analysis for GPA and CD41 of sorted CD144+45− cells grown in haemopoietic medium for 12 and 25 days (day 42 = day 12 in haematopoietic medium, day 55 = day 25 in haematopoietic medium). A representative experiment out of 5 is shown.

Overall, the EPCs, instructed by the haematopoietic medium, are replaced within a few days by haematopoietic cells: notably this happens through an intermediate stage of cell population expressing both haematopoietic and endothelial markers. To formally exclude that our observation derived from expansion of a contaminant hemopoietic subpopulation, we sorted the adherent cells (day 30 of MH-CM culture) by means of CD144 and CD45 markers. Specifically, CD144+45− were sorted using a gate ensuring >99% cell purity (Figure 5A). Purified CD144+45− cells, grown in haematopoietic medium, were analyzed by FACS and confocal analysis for expression of CD144 and CD45 markers (Figure 5B) at different time points, thus monitoring their phenotypic conversion. At a later time (around 12 days of culture in haematopoietic medium) all cells were CD144−45+ that generated first an erythroid and then a megakaryocytic progeny (Figure 5C), as assessed by morphological characterization and immunophenotypic analysis for GPA and CD41 expression (erythroid and megakaryocytic markers, respectively). Taken together, our data indicate that the adherent CD144+45− EPCs function as haemogenic endothelium capable of generating differentiated CD45+ haematopoietic progeny, mostly megakaryocytic and erythroid, through an intermediate transient CD144+45+ developmental stage.

**Figure 6:**
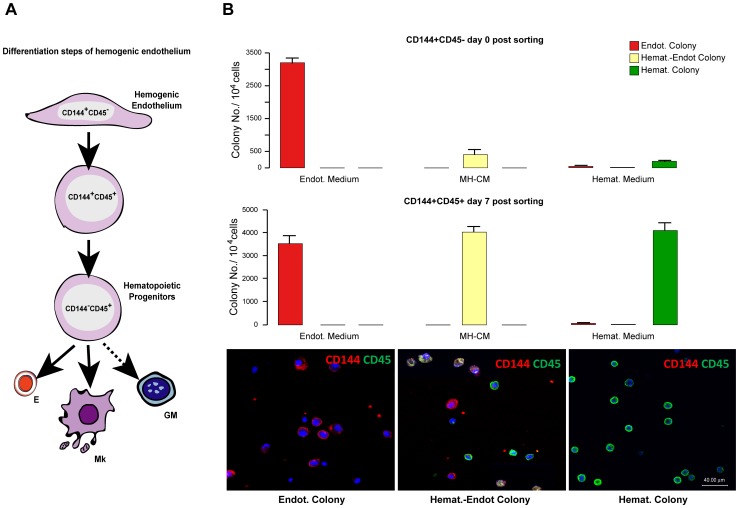
Differentiation of human post-natal haemogenic endotelium into haematopoietic cells. A- Schematic representation of the various stages of haemogenic endothelium differentiation towards haemopoietic cells. B*- Top* Clonogenetic assay of sorted CD144+45− cell populations in unicellular liquid culture in the presence of endothelial, MH-CM and haematopoietic medium. *-Middle* Clonogenetic assay of sorted CD144+45+ cell populations in unicellular liquid culture in the presence of MH-CM and haematopoietic medium. Single cells (at a 0,25 cell/well dilution) were seeded in the different media: the number and the morphology of colonies scored after 20 days. Three independent experiments are reported. - *Bottom*: Confocal double immunofluorescence analysis for CD45 (green) and CD144 (red) antigen expression in endothelial, haemat.-endot. and haematopoietic colonies grown in unicellular liquid culture in the presence of the different media described above.

Finally, we aimed at proving the observed capacity of the adherent CD144+45− to give rise, when instructed by HGFs, to haematopoietic cells at a single cell level. Specifically, we performed a series of limiting dilution of the CD144+45+ cells isolated from the adherent cells endowed by haemogenic endothelium capacity. As control, similar experiments were also performed in parallel on the HGFs-uninstructed cell population (CD144+CD45−). To ensure cell clonality, sorted cells were diluted down to 0.25 cell/well: after their seeding in culture, the wells were inspected by appropriate microscopy examination to ensure that ∼75% of the wells were cell-negative while the ∼25% of positive wells contained a single cell (see Materials and Methods). Single sorted cells were followed by cellular/molecular analysis of their progeny. Unicellular cultures of CD144+45+ cells, within three weeks of culture generated the following colonies: 40% generated colonies displaying a mixed haemato-endothelial phenotype when seeded in MH-CM, 41% produced haematopoietic colonies in the haematopoietic medium and 35% produced endothelial colonies in the endothelial medium. Differently, unicellular cultures of CD144+CD45− cells, gave rise to 33% endothelial colonies (in endothelial medium) but to only a very small number of hematoendothelial colonies (in MH-CM) and hematopoietic colonies (in hemopoietic medium): 4% and 2% respectively (Fig. 6B).

The clonal progenies were also analyzed by combined CD144/CD45 confocal microscopy: cells derived from colonies grown in hematopoietic medium were found CD144 negative and CD45 positive (Figure 6B).

Altogether, these results, summarized in figure 6A, indicate that human CB derived CD34+ cells, instructed by MH-CM to proliferate and acquire the EPC phenotype, function as haemogenic endothelium: they may be reprogrammed by HGFs into the haemopoietic lineages, the latter ones being largely composed of erythroid and megakaryocytic cells.

## Discussion

Recent studies on murine embryos provide evidence that haemangioblasts generate haematopoietic cells/endothelial cells through the haemogenic endothelium [20], [22], [23]. Little is known of the plasticity of endothelial/haematopoietic precursors at later stages of development. Human CB CD34+ cells provide a unique model to investigate the common origin of haematopoietic and endothelial lineages in perinatal life. In fact, these cells represent a standard source for 90−95% HPCs, as well as for a minority of EPCs [24], [25] and early progenitor cells that can be programmed to efficiently generate induced pluripotent stem cells [26]. Building upon our previous work [7], the present studies show that the CB CD34+ cells may be instructed to generate haemogenic endothelium, which in turn, when properly instructed, differentiates into CD45+ CD144+ cells able to differentiate into hemopoietic cells; particularly, we described this multi-step differentiation process at morphological, immunophenotypical and functional levels. Particular attention was devoted to the controls ensuring clonality in the limiting dilution unicellular culture, as detailed above.

Initially, the CB CD34+cells, instructed by MH-CM, acquire an adherent phenotype as well as EPC functional properties. These cells were characterized as CD144+, CD105+, CD146+, CD54+, CD31+, Tie2+, CD49d-, CD43−, CD133− and proven to generate functional vessels *in vivo*. This specific EPC cellular population shows remarkable plasticity, specifically the capacity to express the haematopoietic gene differentiation program upon appropriate instruction. In fact these EPCs, triggered by a HGF cocktail, switch in ∼1-wk into a CD144+45+ progeny, comprising of ∼30–40% functional progenitors capable of giving rise haematopoietic populations. Live imaging of the switch from CD144+CD45− to CD144+CD45+ cells, demonstrated, in line with a previous observation [16], that this transition is not mechanistically linked to mitosis. The frequency of cells capable of haemogenic transition in this cell population is dramatically more elevated than that previously reported by experiments on embryonic stem cells [16], [27], and studies on CB CD34+ cells [7], [8].

After a longer period of time, the HGF cocktail instructs the intermediate, transient CD144+CD45+ cell population to generate a haematopoietic progeny, largely composed of both erythroid and megakaryocytic cells, thus reproducing the haematopoietic cell expression pattern observed in early human development. Notably the CD144+CD45+ haematopoietic progeny display a number of features commonly observed in hemopoietic cells derived from CD34+ cells isolated from cord blood. In particular, erythroid cells express fetal hemoglobin and megakaryocytic cells are mononucleated or scarcely polyploidy [28].

These results raise a number of questions, while offering new and interesting opportunities. Indeed, future studies will be necessary to define the *in vivo* significance of the present observations, specifically of haemogenic endothelium in both postnatal and adult human life. In murine and zebrafish embryo, direct generation of haematopoietic precursors from aortic haemogenic endothelium has been demonstrated [17], [18], [19]. However, other studies also suggest the generation of haematopoietic cells from haemangioblasts in the mesenchymal tissue (reviewed in [29]), or from haemogenic endothelium through haemangioblasts [20].

In our *in vitro* studies on CB human HPCs/EPCs, we described the slow conversion of the CB CD34+ HPC population into haemogenic endothelium, induced by the MH-CM instruction. MH-CM was previously characterized for its capacity to sustain murine and human early progenitor differentiation [21], [30], [31]. While the biological role of specific GFs/GF cocktails present in MH-CM has not yet been elucidated, we provided evidence that CD34+ proliferation and differentiation in endothelial precursors bearing haemogenic potential also require CD34+ cells to release autocrine/paracrine soluble factors [21]. As described, the haemogenic endothelium/haematopoietic interconnection was mechanistically controlled by a multilineage HGF cocktail that rapidly reprograms haemogenic endothelium, first into haematopoietic progenitors, and then into erytrocytic/megakaryocytic cells.

The phenomena described here, together with the embryonic studies mentioned above, highlight the exquisite plasticity of haematopoietic and endothelial primitive cells for interconversion and differentiation, possibly driven *in vivo* by the microenvironment and reproduced *in vitro* through specific GF/cytokine stimuli. While studies on murine embryos suggest that haemogenic endothelium is a transient population linked to specific developmental stages, our data indicate that haemogenic endothelium is not characterized by its transient and exclusive existence in the embryonic period, but rather suggest its existence and, possibly, its functional role throughout human life.

In our view, availability of a purified population of haemogenic endothelium will allow innovative studies at a basic and possibly clinical level. At the clinical level, strategies might be devised to expand the purified haemogenic endothelium *in vitro* in order to explore its potential therapeutic use.

## Materials and Methods

### Cell Purification

Cord blood was obtained from healthy, full-term placentas according to institutional guidelines A.Fa.R. Research Centre, San Pietro Hospital, Fatebenefratelli, Rome, 00100, Italy. The use of human cord blood samples for research pourposes was approved by the Institutional Review Board of the Istituto Superiore di Sanità, Rome, Italy. Low-density mononuclear cells (MNCs) were isolated and CD34+ cells purified as in [7]. The purity of CD34+ cells assessed by flow cytometry was routinely >95%. Each single experiment may included pooled cells derived from different (2/3) cords blood. In some experiments CD144+45− cells were sorted twice to ensure a final purity of >99%, using a fluorescence-activated cell sorter, FACSVantage or FACSAria (Becton-Dickinson).

### Cell Culture

#### Liquid culture. Haemato-endothelial culture

Isolated CD34+ cells were cultivated in MH-CM [21] either in bulk culture (density 1,2–1,5×10^5^ cells/cm^2^) on collagen-coated 24–12–6 well plates or in single cell culture (by limiting dilution, see [7]) in flat 96 well plates. Half MH-CM was replaced with fresh conditioned medium twice a week.

#### Haematopoietic multilineage culture

(see [7]) involved serum-free medium (IMDM, GIBCO) containing delipidated bovine serum albumin (BSA 10 mg/ml), saturated human transferrin (Tf 700 µg/ml), and human low-density lipoprotein (LDL 40 µg/ml), supplemented with a haematopoietic growth factor (HGF) cocktail: flt3 ligand (FLT3) 100 ng/ml, kit ligand (KL) 100 ng/ml, IL-3 10 ng/ml, GM-CSF 10 ng/ml, Tpo 100 ng/ml, Epo 3 U/ml, G-CSF 500 U/ml, M-CSF 250 U/ml, IL-6 10 ng/ml and bFGF 10 ng/ml. (in some experiments VEGF 100 ng/ml was also added to support EC survival/growth) in a fully humidified 5% CO2 5% O2 atmosphere. FLT3, KL, Tpo, G-CSF and bFGF were purchased from PeproTech EC; IL-3, GM-CSF, Epo, M-CSF, IL-6 were provided by R&D System.

#### Endothelial culture

Was performed on collagen-coated wells in standard EGM2 medium (CAMBREX).

### Clonogenic Culture

Clonogenic assays in both bulk and limiting dilution culture were essentially performed as in [7].

#### Bulk culture

Briefly, the adherent and non-adherent cells were plated (1.0 −2.0×10^4^ viable cells/ml) in duplicate dishes in *haematopoietic* culture in serum-free medium supplemented with methylcellulose (Methocult H 4100 Stem cell Technologies, Vancouver BC, CA) containing the multilineage HGF cocktail, BSA, saturated human Tf, and human LDL. The colonies were scored at day 15–20. *Endothelial* culture was performed in EGM2 medium and the colonies were scored at day 20–25. *Haemato-endothelial* culture was carried out in MH-CM: the colonies were scored at day 15–20 and the analysis and characterization of colonies was performed as described. [7].

#### Limiting dilution unicellular culture

As reported [7], the adherent and non-adherent cells were seeded in 96 flat wells at limiting dilutions (down to 0.25 cell/well): the wells were collagen-coated and contained either serum- free medium supplemented by multilineage HGF cocktail, or the EGM2 medium or MH-CM medium. 470 wells were utilized for each experiment. In experiments involving a dilution of 0.25 cell/well, we verified the presence of ∼75% cell-negative wells and the presence of a single cell in the ∼25% positive wells: this analysis was based on direct visual injection of the wells by means of an ad hoc designed EVOS AMG microscope (18421 Bothell-Everett Hwy, Suite 150 Mill Creek, WA 98012), specifically designed for unequivocal analysis of single cell culture wells. In fact, colonies were detected only in some unicellular wells, but never observed in cell-negative ones.

### Cell Analysis

#### Morphology

Cells were harvested at different days of culture, smeared on slides by cytospin centrifugation, and stained with May-Grunwald Giemsa. Cells were observed by conventional light field microscopy.

### Antibodies

The following monoclonal antibodies were used: fluoresceine isothiocyanate (FITC) anti-CD15, anti-CD45, anti-CD56, anti-CD144; allophycocyanine (APC) anti-CD45 and anti-CD117; phycoerythrin (PE) anti-CD13, anti-CD14, anti-CD29, anti-CD34, anti-CD41, anti-CD44, anti-CD45, anti-CD61, anti-CD90, anti-CD105, anti-CD110 (TpoR), anti-CD146, anti-CD147, anti-CD166, anti-CD235a (GPA), anti-HLA-ABC, anti-HLA-DR and anti-GM-CSFRα (CD116) (all from BD Pharmingen). Phycoerythrin anti-VEGF-R2 (KDR), anti-hEpoR, anti-hACE (CD143), anti-CD144 (VE-Cadherin) and anti-Tie-2 (all from R&D Systems).

### Flow Cytometry

Cells were suspended in Ca++ Mg++-free phosphate-buffered (PBS) containing 20% FCS, mouse IgG (40 ug/ml), incubated for 10 minutes on ice, and labelled with fluorochrome –conjugated mAbs for 30 minutes on ice. Cell fluorescence was analyzed with the FACS Canto (Becton Dickinson).

### Immunofluorescence

Cells double-stained with PE anti-CD144 and with FITC anti-CD45 conjugated antibodies, as described above, were fixed with 1% paraformaldehyde and rinsed with PBS, 1% BSA, and 0.1% sodium azide. After washing, cells were rinsed with PBS and spotted on lysine-treated glass slides, mounted on a glass coverslip with SlowFade Gold antifade reagent with DAPI (Invitrogen) and analyzed using a confocal microscope (Leica, Wetzlar, Germany).

### Microarray Analysis

Biotin-labeled cRNA targets were obtained from 100 ng of each RNA sample and hybridized to Human GENE ST 1.0 GeneChip arrays according to Affymetrix standard protocols (Affymetrix, Santa Clara, CA, USA). GeneChip.cel files were analyzed using Partek Genomic Suite 6.4, and further elaborated with GeneSpring 7.0 (Agilent).

### Capillary Tube Formation Assay

Capillary tube formation was assessed as described [32], seeding 2–10^4^ ECs/well in Matrigel supplemented with the EGM-2 medium. After 12 hours, capillary-like formation was observed by microscopy.

### In Vivo Analysis

#### Mice and orthotopic mammary fat pad implantation

Experiments involving animals were done in the animal facilities at IFOM-IEO campus (Milan, Italy), in accordance with the Italian Laws (D.L.vo 116/92 and following additions), which enforces EU 86/609 Directive).

Accordingly to the regulatory requirements, our animal facility is fully authorized by the Italian Ministry of Health (DM N° 65/2007-A) and our project has be notified to the Ministry of Health with ID number 11/09. All surgery was performed under sodium pentobarbital anesthesia, and all efforts were made to minimize suffering. NOD.cg-PrkdcscidIl2rgtm1Wjll (NSG) were kindly donated by Dr. L. Shultz, Female NOD.cg-PrkdcscidIl2rgtm1Wjll (NSG) mice, 6- to 9-week-old, were bred and housed under pathogen-free conditions in our animal facilities (IFOM-IEO campus, Milan, Italy).

MDA-MB-436 triple negative breast cancer cells were purchased from the American Type Culture Collection (ATCC, Manassas, VA, USA) and cultured as suggested by the manufacturer. Prior to injection, tumor cells were trypsin detached, washed twice and resuspended in PBS to a final concentration of 10^6^ cells/13 ml. Cell suspension was then mixed with 5 ml growth factor-reduced Matrigel (BD Biocoat) and 2 ml trypan blue solution (Sigma Aldrich) and maintained on ice until injection. In cases where tumor cells were co-injected with 1×10^6^ endothelial cells, cell suspensions were mixed before the final suspension in Matrigel. Aseptic conditions under a laminar flow hood were used throughout the surgical procedure. Mice (n.5×group) were anesthetized with 0.2% Avertin (Sigma Aldrich), made to lay on their backs and 20 ml of cell suspension in Matrigel were injected directly in the fourth mammary fat pad through the nipple using a Hamilton syringe.

Tumor growth was monitored weekly using digital callipers and tumor volume was calculated using the formula: L × W2/2 = mm3.

### Immunohistochemistry

Immunostaining for anti-human-CD34 (clone BQEND/10, Abcam), and anti-human-CD31 (clone JC70-A, Dako) was performed on serial sections of formalin-fixed and paraffin embedded biopsies with an automated immunostainer (Autostainer, Dako, Glostrup, Denmark), using a commercial detection kit (Dako EnVision Plus-HRP, Dako), according to the manufacturer’s instructions. Antigen retrieval was obtained by placing tissue sections in 0.01 M EDTA buffer at pH 8.0 and underwent three cycles at 90°C in a microwave oven operating at 780 W. Negative control sections were incubated with non-immune mouse serum in place of the specific primary antibodies and consistently lacked any staining. Myeloperoxidases cytochemical staining in blood smears was performed with a commercial kit (Sigma Aldrich) following the manufacturer’s instructions.

Images were acquired with an Aperio’s ScanScope XT digital scanning system.

### Confocal Microscopy

Detection of vessel lumen was studied by confocal microscopy. 10 mmsections were immunostained with human-CD34 (clone BQEND) and an anti-mouse Alexa Fluor® 488 secondary antibody. Images were acquired using a Leica TCS SP5 confocal microscope and sequential Z-stacks were performed using a 63×1.4NA oil immersion objective, zoom 3X, 0.3 µm Z step.
